# Maladie cœliaque de l’adulte révélée par une polysérite

**DOI:** 10.11604/pamj.2017.28.105.10878

**Published:** 2017-10-04

**Authors:** Mounira El Euch, Souha Haddad, Madiha Mahfoudhi, Fethi Ben Hamida, Fatima Jaziri, Khaoula Ben Abdelghani, Sami Turki, Taïeb Ben Abdallah

**Affiliations:** 1 Service de Médecine Interne « A » Hôpital Charles Nicolle, Tunis, Tunisie; 2 Laboratoire de Recherche des Maladies Rénales (LR00SP01), Hôpital Charles Nicolle, Tunis, Tunisie

**Keywords:** Maladie cœliaque, syndrome de malabsorption, polysérite, Celiac disease, malabsorption syndrome, polyserositis

## Abstract

La maladie cœliaque (MC) représente une maladie auto immune touchant plusieurs organes. Elle est souvent révélée par des manifestations digestives avec une malabsorption biologique. Cependant, l’atteinte des séreuses peut exceptionnellement révéler cette entéropathie rendant le diagnostic difficile. Il s’agit du patient JA âgé de 63 ans admis pour exploration de syndrome de malabsorption avec des signes de polysérite à type de pleurésie, péricardite, ascite et hydrocéphalie. Le diagnostic de MC a été porté devant des signes endoscopiques sans arguments sérologiques. L’évolution était partiellement favorable à cause de la mauvaise adhésion au régime sans gluten. Notre observation est le premier cas rapporté d’une polysérite révélant la MC qui devrait être évoquée devant tout syndrome de malabsorption en l’absence de signes évocateurs.

## Abstract (en)

Celiac disease (CD) is an autoimmune disease affecting multiple organs. It often presents as gastrointestinal manifestations associated with malabsorption. However, serosa involvement uncommonly reveals this enteropathy, making the diagnosis difficult. We here report the case of JA, aged 63 years, admitted to hospital to detect the cause of malabsorption syndrome associated with polyserositis signs including pleurisy, pericarditis, ascites and hydrocephalus. The diagnosis of CD was based on endoscopic signs without serology tests. Patient’s evolution was partially favorable, due to lack of compliance with a gluten-free diet. Our study reports the first case of CD revealed by polyserositis. CD should be suspected in patients with malabsorption syndrome, in the absence of evocative signs.

## Introduction

La maladie cœliaque (MC) est une entéropathie inflammatoire chronique auto-immune, se manifestant généralement par une triade comportant une diarrhée, des douleurs abdominales et un syndrome de malabsorption. Cependant des formes sèches ont été rapportées dans la littérature. Nous rapportons l’observation d’un adulte Tunisien pour lequel le diagnostic de MC a été porté à l’âge de 63 ans associant un tableau initial de polysérite avec un syndrome de malabsorption biologique.

## Patient et observation

Il s’agit du patient J.A âgé de 63 ans diabétique, hypertendu admis pour exploration d’un syndrome de malabsorption découvert fortuitement lors d’un bilan biologique de routine fait dans le cadre du suivi de son diabète. Il se plaignait de céphalées occipitales associées à une dyspnée d’effort stade 3 de NYHA. Il n’avait pas de diarrhée ni amaigrissement rapporté. A l’examen, l’auscultation cardiaque était sans anomalies. L’auscultation pulmonaire avait objectivé des râles crépitants à droite. L’examen neurologique avait objectivé un nystagmus horizontal bilatéral. L’examen abdominal était sans anomalies. Le reste de l’examen était sans particularités. A la biologie: Un syndrome de malabsorption était noté avec une anémie hypochrome microcytaire à 10,9 g/dl, une hypo albuminémie à 22g/l, une hypocalcémie (calcémie corrigée à 1,9 mmol/l), une hypocholestérolémie à 1,8 mmol/l. Les bilans rénal et hépatique étaient corrects. La protéinurie de 24h était négative. L’ECG était sans anomalies. La radiographie du thorax avait objectivé un épanchement pleural droit de grande abondance avec collapsus aéré de l’hémi champ pulmonaire droit. La recherche de BK dans les crachats était négative. La ponction pleurale avait conclu à un liquide transudatif (taux de protides à 22 g/l) avec culture bactériologique négative. La biopsie pleurale était négative. Un scanner cérébral avait montré une hydrocéphalie quadri ventriculaire à pression normale. L’échographie trans-thoracique avait montré un épanchement péricardique minime avec une fraction d’éjection conservée. L’échographie abdominale était normale. Un entéroscanner avait objectivé un épanchement intra péritonéal de faible abondance sans anomalies associées des anses jéjunales. La fibroscopie œsogastroduodénale avait objectivé une gastropathie congestive diffuse avec à la biopsie duodénale une atrophie villositaire partielle associé à une lymphocytose intraépithéliale compatible avec une maladie cœliaque stade 3a de la classification de Marsh modifiée. La sérologie de la maladie cœliaque était négative. Le diagnostic de la maladie cœliaque révélé par un tableau de polysérite associé à un syndrome de malabsorption a été retenu. Le patient a été mis sous régime sans gluten et diurétiques. L’évolution était partiellement favorable à cause de la mauvaise observance du régime.

## Discussion

La MC est une pathologie inflammatoire chronique déclenchée par la consommation de gluten contenu dans les céréales [1]. Sa prévalence actuelle est estimée à environ 1% et sa pathogenèse est expliquée par la digestion incomplète de la protéine du gluten sur un terrain génétique parfois prédisposé qui active les lymphocytes T à l’origine de l’atrophie villositaire [1]. Le polymorphisme génétique au cours de la MC a été décrit et on a récemment incriminé le rôle du gène TNF-α-308 G > A dans sa pathogénie [2]. Les manifestations cliniques de la MC sont très diverses et la polysérite est exceptionnellement décrite et n’a jamais été rapportée comme révélatrice de la MC jusque-là. La maladie est révélée généralement de manifestations essentiellement digestives selon une récente cohorte Suisse de 1689 patients avec toutefois 1,8% des patients complètement asymptomatiques [3].

Notre patient présente une forme de MC sèche sans troubles du transit et le diagnostic a été porté sur la fibroscopie digestive haute. Les avancées dans les méthodes de diagnostics et de dépistage ont contribué à une augmentation apparente de l’incidence des MC puisque plus de 80 % des cas diagnostiqués sont asymptomatiques ou pauci symptomatiques [4]. Sa sérologie était négative et on estime qu’elle n’est utile que dans 58%; 44,8% et 36,8% respectivement pour les anticorps anti gliadine de type A, les anti endomysium et les anti transglutaminases [5]. Seul le régime sans gluten permet d’améliorer les symptômes de la maladie qui semble très difficile à respecter avec une moindre efficacité dans les formes réfractaires [6].

## Conclusion

L’atteinte des sérites au cours de la maladie cœliaque a été rarement décrite dans la littérature d’où l’intérêt de notre observation incitant à pratiquer les sérologies spécifiques de cette maladie devant tout tableau de polysérite surtout devant un épanchement de type transudatif.

## Conflits d’intérêts

Les auteurs ne déclarent aucun conflit d’intérêts.
